# Renal Artery Embolization Before Radical Nephrectomy for Complex Renal Tumour: Which are the True Advantages?

**DOI:** 10.1515/med-2019-0095

**Published:** 2019-11-07

**Authors:** Giovanni Cochetti, Michele Del Zingaro, Andrea Boni, Massimiliano Allegritti, Jacopo Adolfo Rossi de Vermandois, Alessio Paladini, Maria Giulia Egidi, Giulia Poli, Pietro Ursi, Roberto Cirocchi, Ettore Mearini

**Affiliations:** 1 Department of Surgical and Biomedical Sciences, Urology Clinic of Perugia, Perugia University, P.le Menghini, 06100, Perugia, Italy; 2 Division of Interventional Radiology, S. Maria Hospital, Terni, Italy; 3 Department of Surgical and Biomedical Sciences, Division of Week surgery, S. Maria Hospital, Terni, Italy; 4 Department of General Surgery Paride Stefanini, Umberto I Policlinico Roma, Italy

**Keywords:** Radical nephrectomy, Embolization, PRAE, Renal masses, Huge mass

## Abstract

**Introduction:**

Renal artery embolization is performed before radical nephrectomy (RN) for renal mass in order to induce preoperative infarction and to facilitate surgical intervention through decrease of intraoperative bleeding. Moreover, in metastatic renal cancer it seems to stimulate tumour-specific antibodies, even if no established benefits in clinical response or survival have been reported. The role of preoperative renal artery embolization (PRAE) in management of renal masses has been often debated and its real benefits are still unclear. Nevertheless, in huge and complex renal masses, which are often characterized by a high and anarchic blood supply and rapid local invasion, radical nephrectomy can be challenging even for skilled surgeons. The aim of this prospective randomized study was to evaluate the effectiveness and safety of PRAE in complex masses by comparing perioperative outcomes of RN with and without PRAE.

**Materials and methods:**

From December 2015 to May 2018 we enrolled prospectively 64 patients who underwent RN for localized (T2a-b) or locally advanced (T3 and T4) or advanced (N+, M+) renal cancers. Patients were divided in two groups. The first group included 30 patients who underwent PRAE; in the second group we enrolled 34 patients who did not undergo RN without PRAE. Perioperative outcomes in terms of operative time, blood loss, transfusion rate and length of hospitalization were evaluated. Statistical analysis was performed using GraphPad Prism 6.0 software.

**Results:**

Median blood loss was 250 ml (50-500) and 400 ml (50-1000) in the first and second group, respectively, with a statistically significant difference (p=0.0066). Median surgical time was 200 min (90-390) and 240 min (130-390) in PRAE and No-PRAE group (p=0.06), respectively. No major complications occurred after embolization. Overall complication rate in Group 1 and 2 was 46.7% (14/30) and 50% (17/34), respectively (p=0.34). No major complications occurred in both groups. The mean follow up was 21,5 months.

**Conclusions:**

Our results prove PRAE to be a safe procedure with low complications rate. To our experience, PRAE seems to be a useful tool in surgical management of a large mass and advanced disease.

## 1 Introduction

Renal cell carcinoma (RCC) represents 3% of all cancers with the highest incidence in Western countries [1]. In 2012, there were approximately 84,400 new cases of RCC and 34,700 kidney cancer-related deaths in the European Union [2]. Radical nephrectomy (RN) is the gold standard treatment for tumours larger than 7 cm as well as for locally advanced and metastatic diseases [3]. In 1973, renal artery embolization was introduced, to clinical practice by Almgard *et al*. to induce necrosis in renal neoplasms. [4] The first indications for renal embolization without nephrectomy were limited to treatment of severe symptomatic hematuria and other palliative strategies for metastatic renal cancer [5, 6]; afterwards, it was performed before RN for renal masses in order to induce preoperative infarction and, consequently, to facilitate surgical intervention through decrease of intraoperative bleeding. Moreover, embolization with delayed RN has been carried out in metastatic RCC with the aim to stimulate tumour-specific antibodies, even if no established benefits in clinical response or survival have been reported [7]. Afterwards, the indications for renal embolization have been extended to different conditions such as persistent bleeding, treatment of hemorrhagic angiomyolipomas (AML), arteriovenous fistulae and vascular malformations, before endograft placement for abdominal aortic aneurysm repair, pseudo-aneurysm, medical renal disease as malignant hypertension and severe nephrotic syndrome [5, 6]. However, the role of preoperative renal artery embolization (PRAE) in management of renal masses has been often debated and its real benefit is still unclear. Nevertheless, in huge and complex renal masses, which are often characterized by high and anarchic blood supply and rapid local invasion, RN can be challenging even for skilled surgeons. To our knowledge, there are no prospective and randomized good quality studies reported in the literature. The aim of this prospective randomized study was to evaluate the effectiveness and safety of PRAE in complex masses comparing perioperative outcomes of RN with and without PRAE.

## 2 Materials and Methods

In a high-volume tertiary institution, a prospective randomized study was carried out to evaluate perioperative data and perioperative complications. The study was approved by the Ethics Committee of the University of Perugia. All subjects signed an informed consent. From December 2015 to May 2018 we prospectively enrolled a total of 64 patients undergoing RN for renal cell cancer (RCC). Patients were randomly assigned to two groups. The first (PRAE group) included patients who underwent PRAE; the second group (No-PRAE group) included patients who did not undergo PRAE. Simple randomization by computer-generated random numbers was performed. Before undergoing RN, abdominal computerized tomography (CT), or magnetic resonance imaging (MRI) in case of kidney failure, were performed in each case. Renal nephrometry score [8] was used to quantify the tumour’s relevant anatomical features as they related to the complexity of the mass, aiding the treatment decision-making. T2 tumours with nephrometry scores of 10-12 were considered high complexity [9] and were included in the study. Other inclusion criteria were locally advanced (T3 and T4) or advanced (N+, M+) renal cancers. Exclusion criteria were T1 masses and bilateral or multi-focal tumours. Heavy BMI or comorbidities were not considered as exclusion criteria. Karnofsky performance status scale [10] and Clavien-Dindo Classification [11] were used to quantify functional status and to evaluate complications, respectively. Laparoscopic surgery was preferred except if minimally invasive approach was technically unsuitable; in these cases a thoracic-phrenic-laparotomy surgery was chosen. The surgical technique did not differ between embolized and non-embolized patients. Lymph node dissection was performed according to the imaging and surgical findings. The endpoint of this study was to evaluate the effect of PRAE on perioperative outcomes in terms of operative time, blood loss, transfusion rate, complications and length of hospitalization. Within the first 2 years after surgery, standard follow-up included physical examination and ultrasound every 3 months as well as CT of the abdomen and thorax every 6 months. After the second year, physical and ultrasound examination was performed every 6 months and CT of the abdomen and thorax once a year. Data were analyzed with GraphPad Prism 6.0. Patient characteristics and perioperative outcomes were analyzed using appropriate comparative tests (T Student, Mann Whitney and Fisher’s exact tests) to verify statistical differences between variables under analysis; the significance threshold was set at 0.05.

### 2.1 Embolization Technique

**Figure 1:**
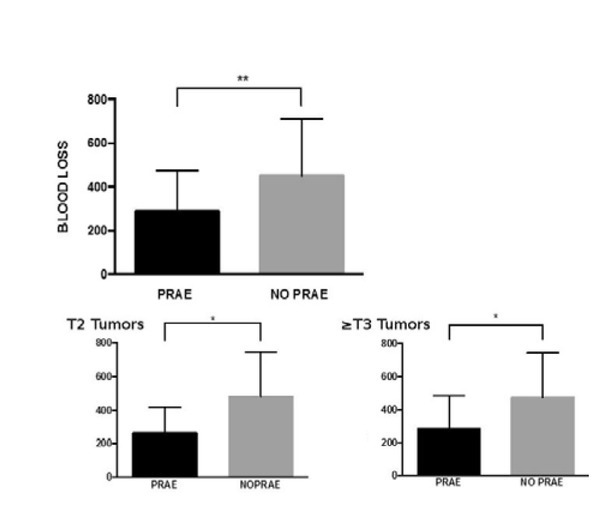
Blood loss report

Renal artery embolization via inguinal percutaneous access through the femoral artery was performed by the interventional radiologist. PRAE was generally performed the day before surgery under local analgesia. After confirmation of the tumour vascularization with a contrast study, the selective renal artery embolization was performed. Different techniques may be used [12]. In our series Haemostatic Absorbable Gelatin Sponge (Spongostan, Ethicon™, Somerville, NJ, USA), Polyvinyl Alcohol (PVA) Embolization particles (Contour, Boston Scientific™, Marlborough, MA, USA), and metallic spirals were preferred (Figure 1). The embolization of accessory renal artery branches was performed only when it was safe and technically feasible. In case of post infarction syndrome (PIS) our treatment consisted of analgesic therapy with paracetamol 1g every 8 hours and, in case of unremitting pain, by a subcutaneous injection of 5 mg of morphine. Antiemetic and antipyretic drugs were used as needed.

## 3 Results

**Figure 2:**
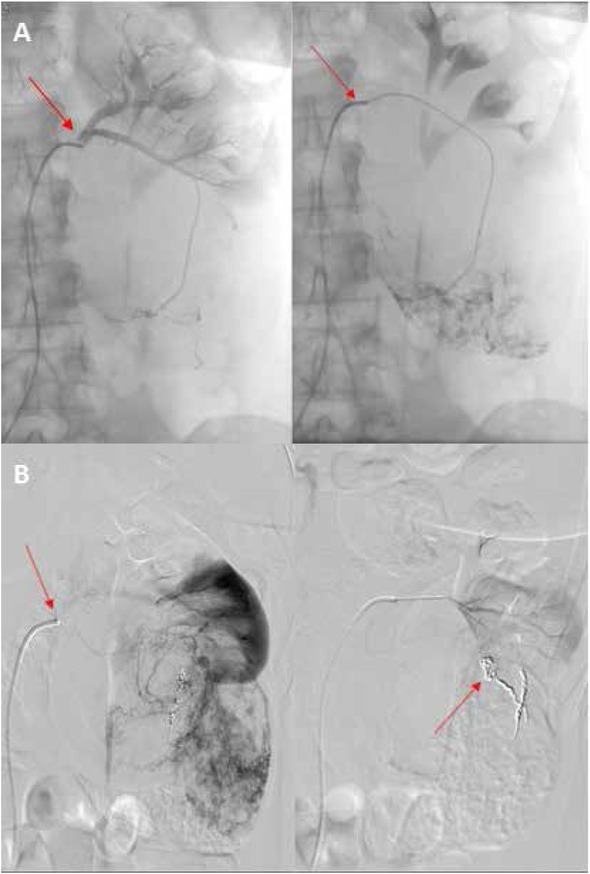
Selective digital subtraction angiography in patient with renal cell carcinoma of left kidney. (A). Postembolization angiography showing complete occlusion of arterial vessels of the left renal mass with Embolization particles (B)

PRAE and No-PRAE group included 30 and 34 patients, respectively. Demographic data were comparable between two groups. Median BMI was 29 kg/m2 (range 20-39) and 28 kg/m2 (range 22-36), respectively for group 1 and 2, median Karnofsky Performance status scale was 90 for both groups. For the PRAE group, median clinical tumour size was 11 cm (ranged from 8 to 17 cm), whereas for the No-PRAE group it was 8.8 cm (ranged from 8 to 16 cm) (p=0.0001). RN was performed after PRAE with a median delay of 21 hrs (ranged from 14 to 30 hrs). PIS occurred in 87% of cases and all of the cases needed pharmacological treatment. No major complications occurred after embolization; there were no cases of coil migration, adjacent organ injury or PRAE-related death. In PRAE group, 12/30 (40%) patients underwent laparoscopic surgery, of which 8 with retroperitoneal approach and 4 with transperitoneal one; the other patients (60%) underwent open trans-peritoneal RN. Lymphadenectomy was carried out in 18/30 (60%) patients. In No-PRAE group, laparoscopic transperitoneal approach was used in 18/34 patients (53%), open surgery in 16/34 (47%); lymphadenectomy was performed in 56% of cases (19/34). Clinical and pathological patients characteristics as well as perioperative

data are showed in Table 1. For the PRAE group, median blood loss was 250 ml (50-500 ml) while for No-PRAE group was 400 ml (50-1000 ml) (Figure 2). Blood loss was significantly higher in No-PRAE group than in PRAE group (p=0.0066). However, no difference was found for transfusion rate (p>0,05). Median surgical time was 205 min (ranged from 90 to 390 min) for the PRAE group versus 240 min (ranged from 130 to 390 min) for the No-PRAE group with no statistically significant difference (p=0.06). Blood loss and surgical time were then compared between the two groups, stratifying according to clinical staging (T2 vs≥T3 lesions). PRAE group showed significantly lower blood loss than No-PRAE group both in T2 and ≥T3 clinical stage (p=0.03) [Figure 1]. On the contrary, surgical time did not differ between T2 (214 min vs 228 min; p=0.54) and ≥T3 lesions (185 min vs 243 min; p=0.08). Median length of hospital stay was 8 days for both groups. This result did not statistically differ (p=0.37) neither by stratifying T2 (p=0.36) from ≥T3 (p=0.25). Overall complication rate was

**Table 1:** Demographic, clinical and pathological data

Characteristic	Total Cohort	%	PRAE group pt.	%	No-PRAE group pt.	%
Patients n	64	100%	30	47%	34	53%
Sex Male	29	45.3%	11	37%	18	53%
Female	35	54.7%	19	63%	16	47%
Age Median	67.5		65.7		66.2	
Range	19-81		19-81		38-80	
Site Right	30	46.8%	14	46.6%	16	47%
Left	34	53.2%	16	53.4%	18	53%
Median Clinical Size (range)	10 cm (8-17)		11 cm (8-17)		8 cm (8-16)	
PNS						
Anemia	19	29.6%	9	30%	10	34%
Fever	20	31.2%	8	66%	12	35%
Asthenia	13	20.3%	7	23%	6	18%
Weight Loss	9	14%	4	13%	5	14%
Hypertension	3	4.7%	3	10%	0	0%
Ascites/Aedema	2	3.1%	1	3%	1	3%
cT Staging						
cT2b	23	36%	11	37%	11	33%
cT3a	27	42%	16	53%	12	35%
cT3b	9	14%	0	0%	9	26%
cT4	5	7%	3	10%	2	6%
cN Staging						
cN0	29	44%	12	40%	15	45%
cN1	27	42%	14	47%	14	41%
cN2	9	14%	4	13%	5	14%
cM Staging						
cM0	41	64%	20	67%	21	62%
cM1	23	36%	10	33%	13	38%
pT Staging						
pT0	6	9%	2	7%	4	12%
pT2	17	26%	11	37%	4	35%
pT3	40	63%	16	53%	26	59%
pT4	1	2%	1	3%	0	0%
Grading						
G0	6	9%	2	7%	4	12%
G1	4	6%	2	7%	1	3%
G2	18	28%	6	20%	10	29%
G3	30	47%	16	53%	14	41%
G4	9	14%	4	13%	5	15%
pN Staging						
pNx	16	25%	9	30%	7	20%
pN0	37	58%	14	47%	23	68%
pN1	11	17%	7	23%	4	12%
Margins						
R0	59	92%	28	93%	31	91%
R1	5		2	7%	3	9%
		8%				
PRAE material						
Gelatine Sponge	26	41%	26	87%	n.a	n.a
Polyvinyl Alchool	12	19%	12	40%	n.a	n.a
Metallic Spirals	12	19%	12	40%	n.a	n.a
PIS	26/30	87%	26	87%	n.a	n.a
Median Time P-t-S (range)	21 h		21 h (14-30)		n.a	
	(14-30)					
Histotype						
RCC	50	78.2%	22	74%	28	82%
Oncocytoma	3	4.7%	1	3%	2	6%
Cromophobe	4	6.3%	3	10%	1	3%
Papillary	1	1.5%	1	3%	0	0%
Solitary Fibrous Tumor	1	1.5%	1	3%	0	0%
KS	4	6.3%	2	7%	2	6%
TCC	1	1.5%	0	0%	1	3%
Median Blood loss (range)	325 cc (50-1000)		250 cc (50-500)		400 cc (50-1000)	
Median Surgical Time (range)	220 min (90-390)		205 min (90-390)		240 min (130-390)	
Transfusion Rate	23/64	36%	9	30%	14	41%
Intraoperative Transfusion Rate	13/64	20%	4	13%	9	26%
Hospital Transfusion Rate	17/64	26%	7	23%	10	29%
Median Hospital Stay (range)	8 d (5-20)		8 d (6-20)		8 d (5-15)	
Int. Care Unit stay	12	19%	8	27%	4	12%

Abbreviation: PNS= para neoplastic syndrome; PIS=post infarction Syndrome; P-t-S= PRAE to Surgery; RCC= renal cell carcinoma; SFT= Solitary Fibrous Tumor; KS= Kidney’s Sarcoma; TCC= transitional cell carcinoma.

46.7% (14/30) and 50% (17/34) in group 1 and 2, respectively, without any statistically significant difference (p=0.34). No major complication occurred in both groups. The mean follow up was 21,5 months. Finally, the cancer free survival between the two groups had similar results, which were not reported due to the small cohort and the too short follow up.

## 4 Discussion

PRAE has been introduced to reduce the risk of oncological spread and intraoperative bleeding, in order to facilitate surgery, thus decreasing perioperative morbidity. Nevertheless, in recent literature the real usefulness of PRAE is still debated. Some authors agree that the advantages of PRAE are to decrease intraoperative bleeding with a lower transfusion rate and to reduce operative time [13, 14, 15, 16].

However, most of the studies concerning blood loss from nephrectomy after PRAE are small and non-randomized [18, 19, 20]. We deem that PRAE facilitates RN by reducing the tumour blood supply and therefore, the operative blood loss and blood transfusion requirements; thereby, the operative time may be decreased too, especially in huge and complex renal mass or in tumours with wide blood supply. Other important benefits of PRAE include the potential role of an early ligation of the renal vein before the renal artery has been fully controlled, according to the indications given by Robson [21*]*. He suggests the renal artery ligation before the vein in order to reduce the oncological spread due to tumour manipulation [22]. This surgical strategy may be really advantageous in case of huge renal mass with anarchic vascular growth or whenever there is a perihilar disease or in a presence of hilar adenopathy, alleviating some of the technical surgical difficulties. Our prospective, randomized study demonstrated that PRAE is a safe and well tolerated procedure with a low, even if not negligible, complications rate.

We found a significant decrease of median blood loss in patients that underwent PRAE (250 ml vs 400 ml, p=0.0066). This finding highlighted one of the most relevant advantages of PRAE, in particular in case of RN for huge and complex mass. Likewise, in our series the surgical time in PRAE group has been reduced (median surgical time was 205 minutes versus 240 minutes) even if the difference is not statistically significant especially for the T2 lesions. Also for T3 tumour a significant difference has not been proved, but the results are quite close reflecting a general aid in the technical difficulties of surgery for the larger masses. In 2014, Zargar et al. reported their results from performing PRAE in locally advanced kidney cancer; moreover, they reviewed the recent literature comparing several studies with different results (Table 2). The authors concluded that PRAE could reduce overall post-operative complications rate (42%), intraoperative blood loss (750cc) and blood transfusion rate [23]. However their findings about blood loss and transfusion rate were higher than our data; this difference could be due to inclusion in their series of inferior vena cava thrombus tumours that seemed to worsen median duration of surgery and blood loss. Likewise, different experiences in recent literature

**Table 2:** Review of recent literature data

	Our Series		Zargar et al.		Subramanian	et al.	Schwartz
	PRAE	No PRAE	PRAE	IVC PRAE	PRAE	No PRAE	PRAE
Number of cases	30	34	42	15	135	90	66
Median surgical Time (min)	205	205	192.5	258.5	390	313	n.a
Median Transfusion (U)	0	0	2	2	8	4	3.9
Median blood loss (cc)	250	400	750	1550	2000	1500	725
Post-op Complications %	53.3%	44%	45.2%	53%	58.43%	26.29%	n.a
Median Hospital Stay (days)	8	8	9	9	19	10	n.a
Mortality %	0	0	0	0	17.13%	3.3%	n.a

did not clarify if PRAE may improve surgical performance in large masses RN with or without IVC thrombus. We do not usually perform embolization of IVC tumours because venous hypertension reduces significantly the arterial renal flow, erasing potential advantages of PRAE.

Many authors reported minimal morbidity in PRAE due to rare but undeniable detriments as hematoma of the access site, infarction of unpredictable sites as opposite side kidney, bowel, medullar ischemia and vascular lesions, contrast nephrotoxicity, besides other serious complications as coil migrations which are rarely described [16, 17, 22, 23, 24, 25, 26, 27, 28]. Moreover, the risk of failure has to be considered. In our 30 patients who underwent PRAE, complication rate is comparable with patients who did not undergo preoperative embolization. Anyway, complications did not modify the surgical strategy and there were no major complications according to the Clavien-Dindo Classification [6]. The delay to surgery after PRAE is reported from a few hrs to 48 hrs, until several days; RN has been reported being performed also 78 days after PRAE [5]. In our series median time to surgery is about 21 hrs, with a range from 14 to 30 hrs. Timing of 24-48 hrs is recommended because of the risk of emphysematous pyelonephritis that may occur if nephrectomy is performed more than 4 days after renal artery embolization. The renal infarction may cause a post-infarction syndrome (PIS) in almost 90% of patients [24]. Symptoms as flank pain, fever, nausea and vomiting with or without paralytic ileus are the classic presentations of PIS. In our series, PIS occurred in 87% of cases (26/30) and was treated by paracetamol 1g every 8 hours and, in more severe cases, by 5 mg morphine injection on demand. Septic complications of post-infarction syndrome can reach as high as 10% and they depend strictly on the timing of surgery after PRAE [25]. It should be pointed out that the lack of post-infarction syndrome might be indicative of an incomplete embolization due to a collateral vascular circle or a failure of the hemostatic material placement. However, in our study 4 patients did not show PIS: 2 cases showed an efficient embolization and only 2 cases had a series of collateral vessels that did not consent a complete ischemia. Lin et al. reported renal embolization simultaneous to nephrectomy in order to avoid all the complications arising from the waiting time between the two procedures [26]*. Although* performing both procedures concomitantly in the same surgical act appears to retain the advantages of the PRAE, we preferred to wait about 24 hrs in order to warrant an optimal stationary arrangement of the embolization.

Finally, PRAE also may induce immune response against the tumour. Nakano et al. reported a direct role of the embolization in the modulation of the immune lymphocyte proliferative response and Bakke et al. confirmed an implementation of the natural killer cell activities after embolization [27, 28]. In a retrospective study Zielinski et al. [29] compared 118 patients who underwent PRAE before nephrectomy with a case-matched control group including 116 patients who underwent nephrectomy alone. They found a significant survival benefit in the PRAE group. Nevertheless, this survival benefit applied only to patients with pT2 and pT3 disease and to patients with pT3N+ at the time of surgery. However, these observations are not univocally confirmed in recent literature and the direct role on the overall survival has to be confirmed by prospective and larger cohort studies with a longer follow-up. Even if affected by too short follow up, our data seem to outline a better trend for the PRAE group, but without reaching a statistically significant value. The main limitation of this study was the small sample size and short follow up.

## 5 Conclusion

The prospective randomized study showed PRAE to be a safe procedure with relatively low complications rate. To our experience, PRAE seemed to be a useful tool in surgical management of huge mass and advanced disease. However further prospective studies with larger sample size and longer follow up are necessary to confirm our results.

## List of Abbreviations

PRAE: Preoperative Renal Artery Embolization
RN: Radical Nephrectomy
RCC: Renal Cell Carcinoma
CT: Computed Tomography
MRI: Magnetic Resonance
AML: Angiomiolypomas
PIS: Post Infarction Syndrome
IVC: Intra Vena Cava
PNS: Para-NeoplasticSyndromes
P-t-S: PRAE-to-Surgery
SFT: Solitary Fibrous Tumour
KS: Kidney Sarcoma
TCC: Transitional Cell Carcinoma
